# Sex-related differences in the applicability and performance of the Montreal Cognitive Assessment in the acute phase of stroke

**DOI:** 10.1093/esj/aakaf019

**Published:** 2025-12-11

**Authors:** Giuseppe Scopelliti, Francesco Mele, Federica Vittoria Ruggiero, Ilaria Cova, Federico Masserini, Valentina Cucumo, Giorgia Maestri, Alessia Nicotra, Arianna Forgione, Pierluigi Bertora, Simone Pomati, Emilia Salvadori, Leonardo Pantoni

**Affiliations:** Neurology Unit, Luigi Sacco University Hospital, Milan, Italy; Neurology Unit, Luigi Sacco University Hospital, Milan, Italy; Neuroscience Research Center, Department of Biomedical and Clinical Sciences, University of Milan, Milan, Italy; Neurology Unit, Luigi Sacco University Hospital, Milan, Italy; Neuroscience Research Center, Department of Biomedical and Clinical Sciences, University of Milan, Milan, Italy; Department of Radiology and Nuclear Medicine, Vrije Universiteit Amsterdam, Amsterdam UMC Location VUmc, Amsterdam, The Netherlands; Neurology Unit, Luigi Sacco University Hospital, Milan, Italy; Neurology Unit, Luigi Sacco University Hospital, Milan, Italy; Neurology Unit, Luigi Sacco University Hospital, Milan, Italy; Neurology Unit, Luigi Sacco University Hospital, Milan, Italy; Neuroscience Research Center, Department of Biomedical and Clinical Sciences, University of Milan, Milan, Italy; Neurology Unit, Luigi Sacco University Hospital, Milan, Italy; Neuroscience Research Center, Department of Biomedical and Clinical Sciences, University of Milan, Milan, Italy; Neurology Unit, Luigi Sacco University Hospital, Milan, Italy; Neuroscience Research Center, Department of Biomedical and Clinical Sciences, University of Milan, Milan, Italy; Department of Neurorehabilitation Sciences, Casa di Cura Igea, Milan, Italy

**Keywords:** acute stroke, MoCA, cognitive assessment, vascular cognitive decline

## Abstract

**Introduction:**

Early cognitive screening, although recommended, can be challenging in acute stroke settings. In patients with acute stroke, we aimed to evaluate (1) reasons and predictors of non-applicability of the Montreal Cognitive Assessment (MoCA) and (2) MoCA score performance, focusing on sex-related differences.

**Patients and methods:**

We conducted a single-centre study on patients consecutively admitted to our stroke unit (June 2019–June 2023). Reasons for MoCA non-applicability and MoCA scores were compared between sexes. Univariate and multivariable analyses explored associations between MoCA applicability and sociodemographic/clinical characteristics.

**Results:**

Out of 637 admitted patients (median age 78.8 years; 54.3% male; 81.2% ischemic stroke), 445 (69.8%) completed the MoCA (76.3% of males, 62.2% of females, *P* <.001). Reasons for non-applicability were acute stroke-related in 63.5% of cases (mainly altered consciousness and aphasia), prestroke conditions-related in 22.9% and other (refusal/unreported) in 13.5%. Stroke-related reasons were more frequent in females (*P* =.002) and refusal in males (*P* =.005). Variables associated with MoCA non-applicability were: NIHSS on admission in both females (adjusted odds ratio [adj.OR] 1.25, 95% CI, 1.16–1.34) and males (adj.OR 1.24, 95% CI, 1.14–1.34); pre-stroke mRS in females (adj.OR 1.58, 95% CI, 1.15–2.17) and years of education and left-hemisphere lesion in males (adj.OR 0.91, 95% CI, 0.84–1.00 and adj.OR 2.38, 95% CI, 1.16–4.86, respectively). Among tested patients, females showed lower raw and adjusted MoCA scores (*P* <.001 and *P* =.022, respectively).

**Conclusion:**

Sex-specific factors influence feasibility and interpretation of early cognitive screening in the acute stroke phase: recognising these differences might guide future efforts towards more inclusive and individualised protocols.

## Graphical Abstract

**Graphical abstract:**
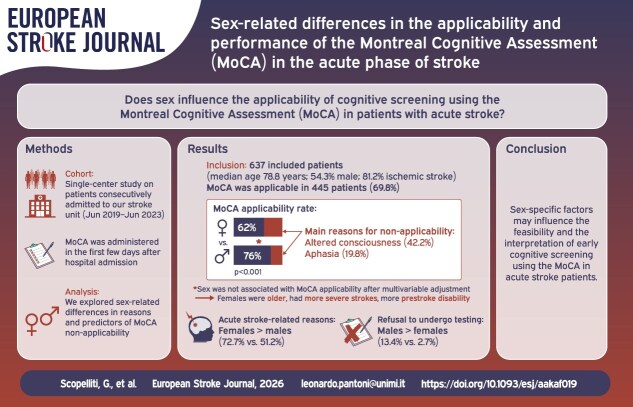

## 1. Introduction

Post-stroke cognitive impairment is a common and significant long-term consequence of acute cerebrovascular events, affecting up to 60% of stroke survivors and severely impacting the quality of life of both patients and caregivers.^1^^,^^2^ Early cognitive assessment can predict long-term cognitive trajectories of stroke patients, supporting the planification of treatment and rehabilitation programs, and guiding discharge planning and home care arrangements.^3–6^ The Montreal Cognitive Assessment (MoCA) is one of the most widely used tools for cognitive screening in stroke patients and offers a quick and comprehensive evaluation of multiple cognitive domains, including attention, memory, language and executive functions.^7^^,^^8^ Despite it being increasingly recommended, cognitive screening in the acute phase can be challenging, with a relevant share of hospitalised patients unable to undergo testing, primarily due to aphasia or impaired consciousness.^9–11^ In this regard, sex-related differences in the applicability and interpretation of cognitive screening in the acute stroke setting are not well understood, but they can be hypothesised based on variations in social factors, demographics, risk factors and stroke trajectories between sexes.^12–14^

In this study assessing the overall applicability and performance of the MoCA in patients with acute stroke, we investigated whether sex differences play a role in this context.

## 2. Patients and methods

### 2.1. Study design and patient inclusion

We retrospectively analysed prospectively collected data of patients admitted to the stroke unit of the Luigi Sacco Hospital in Milan, Italy, between June 2019 and June 2023. All consecutive patients admitted for acute cerebrovascular events were included, with no exclusion criteria based on age, sex, comorbidities, or stroke type. Admissions during the peaks of the COVID-19 pandemic (from March to August 2020 and from November 2020 to October 2021) were stopped due to a reorganisation of regional hospital services. All patients underwent brain imaging during hospitalisation with CT; MRI was added if required as per clinical practice. The study protocol and design have been described in detail in previous publications.^6^^,^^15^^,^^16^

### 2.2. Clinical data

Demographic and clinical variables such as age, sex and education level, along with vascular risk factors, were recorded. The type of acute cerebrovascular event was categorised into ischemic stroke, TIAs, intracerebral haemorrhage or other stroke types (which included cerebral venous thrombosis, subarachnoid haemorrhage, dural fistula and transient focal neurological episodes). Ischemic strokes were further classified into four subtypes: atherosclerotic, cardioembolic, small vessel occlusion and other (the latter category included patients with cryptogenic strokes, multiple identified causes, other uncommon etiologies, or incomplete diagnostic work-ups).^17^ Stroke severity was assessed using the NIHSS. Pre-stroke functional status was evaluated using the mRS.^18^ Two trained neuropsychologists administered the 16-item Informant Questionnaire on Cognitive Decline in the Elderly to informants to assess pre-stroke cognitive status, with a score > 3.3 indicating premorbid cognitive impairment.^19^ Cognitive status during the acute stroke phase was evaluated using the MoCA, with scores ranging from 0 to 30.^8^ The MoCA was administered by trained neurologists upon clinical stabilisation, typically between the second and fifth day of hospitalisation. Age- and education-adjusted MoCA scores were calculated.^20^ Patients were divided into two groups: those who completed the MoCA during their hospital stay and those for whom the MoCA was deemed inapplicable. For the purposes of this study, MoCA was considered applicable when the assessment could be initiated and at least partially completed, regardless of whether all items were administered. In contrast, cases in which the MoCA could not be initiated due to clinical or other constraints were classified as non-applicable. For patients who were not tested, the primary reason for non-applicability was recorded and categorised as follows:

1. *acute-stroke related*, that is, early in-hospital death, altered consciousness, severe motor impairment (eg, complete paralysis in the dominant hand, anarthria), aphasia (with or without severe motor impairment);
2. *pre-stroke conditions*, that is, language barrier, severe cognitive impairment, motor or sensory impairment (eg, blindness, deafness and speech impairment);
3. *other*, that is, patient refusal, reason not reported.

### 2.3. Statistical analyses

Descriptive statistics were used to summarise patients’ characteristics, clinical outcomes and MoCA applicability. Continuous variables were expressed as medians with IQRs or means with SDs, depending on the data distribution, and categorical variables as frequencies and percentages. Between-group comparisons were conducted using the Mann–Whitney *U* test for continuous variables and the Chi-square test for categorical variables. Among patients who were not tested, the reasons for MoCA non-applicability were compared between females and males. To identify predictors of MoCA applicability, a multivariable logistic regression model (*Model 1*) was developed for the entire sample, incorporating clinically relevant variables selected a priori as covariates: age, sex, pre-stroke cognitive status, pre-stroke mRS, NIHSS score on admission, education level and the presence of a left hemispheric lesion. Covariates were selected a priori based on clinical relevance and prior evidence of association with MoCA applicability in acute stroke.^11^^,^^21^ We also performed a sequential logistic regression model using MoCA non-applicability as outcome variable, including sex as a fixed covariate and progressively adding other covariates. Similar models (incorporating clinically relevant variables selected a priori as covariates, excluding sex) were applied separately to female (*Model 2*) and male patients (*Model 3*) to identify potentially different patterns of predictors. Sensitivity analyses of the multivariable models were conducted excluding the small subset of patients (*n* = 12) for whom the reason for MoCA non-applicability was unreported. We assessed multicollinearity using the variance inflation factor, adopting an alert threshold of 2.5, and found no evidence of relevant multicollinearity. Raw and adjusted MoCA scores were compared between females and males using the Mann–Whitney *U* test; a sensitivity analysis excluding patients with partially completed MoCA assessments was then performed. A *P*-value <.05 was considered statistically significant for all tests. All statistical analyses were performed using SPSS (version 27.0).

### 2.4. Standard protocol approvals, registrations and patient consents

All patients provided informed consent for participation in clinical assessments, including neuroimaging. The local institutional review board subsequently approved the retrospective data analysis. All procedures were conducted in accordance with the Declaration of Helsinki. The corresponding author has full access to the study data and is responsible for its integrity and analysis. The data supporting the findings of this study are available from the corresponding author upon reasonable request. This article adheres to the guidelines of the Strengthening the Reporting of Observational Studies in Epidemiology.^22^

## 3. Results

### 3.1. Study cohort

A total of 637 patients were included in the study. Most patients had ischemic strokes (81.2%), followed by intracerebral haemorrhage (10.1%), TIAs (7.1%) and other types of cerebrovascular events (1.6%). The median age of the study population was 78.8 years (IQR 67.6–85.1), 346 (54.3%) were males (Table 1). Females, compared to males, were older (median age 82.3 vs 75.0, *P* <.001), had higher NIHSS scores on admission (median score 5 vs 3, *P* <.001), more pre-event disability defined as an mRS score > 2 (23.8% vs 12.8%, *P* <.001), and higher rates of pre-stroke cognitive impairment (41.1% vs 26.3%, *P* <.001). Table 2 provides a comprehensive overview of the differences between female and male patients.

**Table 1:** Clinical characteristics of patients who were tested and not tested with the MoCA during the acute phase of stroke

	**All patients** ***N =* 637**	**Patients tested with MoCA** ***N =* 445**	**Patients not tested with MoCA** ***N =* 192**	***P*-value **
***Clinical variables***				
Age (years)	78.8 (67.6–85.1)	77.1 (66.1–84.0)	82.6 (72.9–88.0)	**<.001**
Male sex	346 (54.3)	264 (59.3)	82 (42.7)	**<.001**
Years of education, *mean* (*SD*) [*N* = 65 missing]	9.7 (4.7)	9.9 (4.7)	8.8 (4.8)	**.004**
Hypertension [*N* = 4 missing]	485 (76.6)	333 (75.2)	152 (80.0)	.188
Diabetes [*N* = 5 missing]	171 (27.1)	108 (24.4)	63 (33.3)	**.020**
Dyslipidemia [*N* = 11 missing]	383 (61.2)	275 (62.8)	108 (57.4)	.209
Atrial fibrillation [*N* = 3 missing]	142 (22.4)	71 (16.0)	71 (37.2)	**<.001**
Pre-stroke mRS score [*N* = 3 missing]	0.0 (0.0–2.0)	0.0 (0.0–1.0)	0.5 (0.0–3.0)	**<.001**
Pre-stroke cognitive impairment [*N* = 67 missing]	188 (33.0)	122 (28.7)	66 (45.5)	**<.001**
***Acute stroke features***				
NIHSS score on admission [*N* = 15 missing]	4.0 (1.0–8.0)	2.0 (1.0–5.0)	10.0 (4.0–17.0)	**<.001**
Left hemisphere lesion	301 (47.3)	188 (42.2)	113 (58.9)	**<.001**
Cerebrovascular event type				
*Ischemic stroke*	517 (81.2)	362 (81.3)	155 (80.7)	.855
*TIA*	45 (7.1)	38 (8.5)	7 (3.6)	**.027**
*Intracerebral haemorrhage*	65 (10.2)	35 (7.9)	30 (15.6)	**.003**
*Other type*	10 (1.6)	10 (2.2)	0 (0.0)	**.036**
***Ischemic stroke features (N = 517)***				
Ischemic stroke subtype				
*Atherosclerotic*	110 (21.3)	80 (22.1)	30 (19.4)	.485
*Cardioembolic*	159 (30.8)	95 (26.2)	64 (41.3)	**<.001**
*Small vessel occlusion*	51 (9.9)	43 (11.9)	8 (5.2)	**.019**
*Other*^a^	197 (38.1)	144 (39.8)	53 (34.2)	.231
Intravenous thrombolysis	86 (16.6)	59 (16.3)	27 (17.4)	.754
Mechanical thrombectomy	53 (10.3)	23 (6.4)	30 (19.4)	**<.001**

^a^Other ischemic stroke subtype encompasses patients with cryptogenic strokes, multiple causes, other identified cause, incomplete evaluation.

Continuous variables are expressed as “median (interquartile range),” while categorical variables are expressed as “absolute frequency (relative frequency),” except where indicated. Univariate analyses were performed using the Mann–Whitney *U* test for continuous variables and the Chi-square test for categorical variables. Bold values represent statistically significant differences.

Abbreviations: mRS = modified Rankin Scale; pre-stroke cognitive decline = IQCODE score >3.3; IQCODE = Informant Questionnaire for Cognitive Decline in the Elderly; NIHSS = National Institutes of Health Stroke Scale; IQR = interquartile range; SD = standard deviation; TIA = transient ischemic attack.

**Table 2:** Clinical characteristics of female and male patients

	**All patients** ***n = 637***	**Females** ***n = 291***	**Males** ***n = 346***	***P*-value **
***Clinical variables***				
Age (years)	78.8 (67.6–85.1)	82.3 (73.7–87.6)	75.0 (63.6–82.8)	**<.001**
Years of education, *mean* (*SD*) [*N* = 65 missing]	9.7 (4.7)	8.4 (4.5)	10.7 (4.6)	**<.001**
Hypertension [*N* = 4 missing]	485 (76.6)	231 (80.2)	254 (73.6)	.051
Diabetes [*N* = 5 missing]	171 (27.1)	65 (22.6)	106 (30.8)	**.020**
Dyslipidemia [*N* = 11 missing]	383 (61.2)	168 (59.2)	215 (62.9)	.343
Atrial fibrillation [*N* = 3 missing]	142 (22.4)	86 (29.8)	56 (16.2)	**<.001**
Pre-stroke mRS score [*N* = 3 missing]	0.0 (0.0–2.0)	0.0 (0.0–2.0)	0.0 (0.0–0.0)	**<.001**
Pre-stroke cognitive impairment [*N* = 67 missing]	188 (33.0)	106 (41.1)	82 (26.3)	**<.001**
***Acute stroke features***				
NIHSS score on admission [*N* = 15 missing]	4.0 (1.0–8.0)	5.0 (2.0–13.0)	3.0 (1.0–6.0)	**<.001**
Left hemisphere lesion	301 (47.3)	134 (46.0)	167 (48.3)	0.577
Cerebrovascular event type				
*Ischemic stroke*	517 (81.2)	238 (81.8)	279 (80.6)	.711
*TIA*	45 (7.1)	16 (5.5)	29 (8.4)	.157
*Intracerebral haemorrhage*	65 (10.2)	34 (11.7)	31 (9.0)	.258
*Other type*	10 (1.6)	3 (1.0)	7 (2.0)	.358
***Ischemic stroke features (N = 517)***				
Ischemic stroke subtype				
*Atherosclerotic*	110 (21.3)	38 (16.0)	72 (25.8)	**.006**
*Cardioembolic*	159 (30.8)	92 (38.7)	67 (24.0)	**<.001**
*Small vessel occlusion*	51 (9.9)	23 (9.7)	28 (10.0)	.888
*Other*^a^	197 (38.1)	85 (35.7)	112 (40.1)	.301
Intravenous thrombolysis	86 (16.6)	39 (16.4)	47 (16.8)	.889
Mechanical thrombectomy	53 (10.3)	34 (14.3)	19 (6.8)	**.005**

^a^Other ischemic stroke subtype encompasses patients with cryptogenic strokes, multiple causes, other identified cause, incomplete evaluation

Continuous variables are expressed as “median (interquartile range),” while categorical variables are expressed as “absolute frequency (relative frequency),” except where indicated. Univariate analyses were performed using the Mann–Whitney *U* test for continuous variables and the Chi-square test for categorical variables. Bold values represent statistically significant differences.

Abbreviations: mRS = modified Rankin Scale; pre-stroke cognitive decline = IQCODE score > 3.3; IQCODE = Informant Questionnaire for Cognitive Decline in the Elderly; NIHSS = National Institutes of Health Stroke Scale; IQR = interquartile range; SD = standard deviation; TIA = transient ischemic attack.

### 3.2. Acute phase cognitive screening and reasons for non-applicability

**Figure 1:**
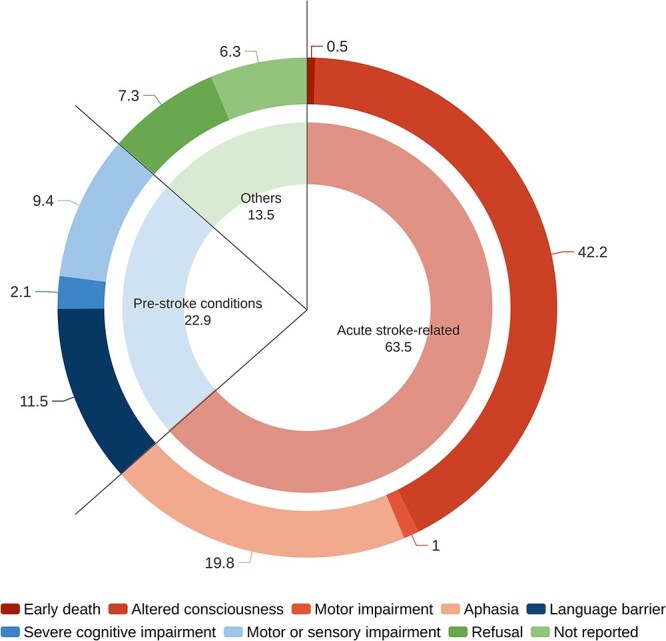
Reasons for non-applicability of MoCA in the acute phase of stroke. The chart depicts the 192 patients who were deemed not testable. Numbers in the chart represent percentages. Abbreviation: MoCA = Montreal Cognitive Assessment.

The MoCA was administered to 445 out of 637 admitted patients (69.8%). In the remaining 192 patients (30.2%) the test was deemed inapplicable. Of the 445 patients who underwent cognitive screening, the MoCA questionnaire was fully completed in 376 patients (84.5%) and partially completed in 69 patients (15.5%). The rate of partial completion was higher in females than in males (22.1% vs 11.0%, *P* =.001). In patients who underwent cognitive screening, the MoCA items most commonly left uncompleted were the alternating trail making test (13.2%), cube copy (10.3%), clock drawing test (9.2%) and verbal fluency (3.1%). The median time from admission to MoCA administration was 3 days (IQR 2–5). The main reasons for not administering the MoCA (Figure 1) were altered consciousness (42.2%), aphasia with or without associated motor impairment (19.8%) and language barrier (11.5%). Among the patients who were not tested, 7.3% refused to undergo the assessment. A reason for non-applicability was not reported in 6.3% of non-tested patients.

**Figure 2:**
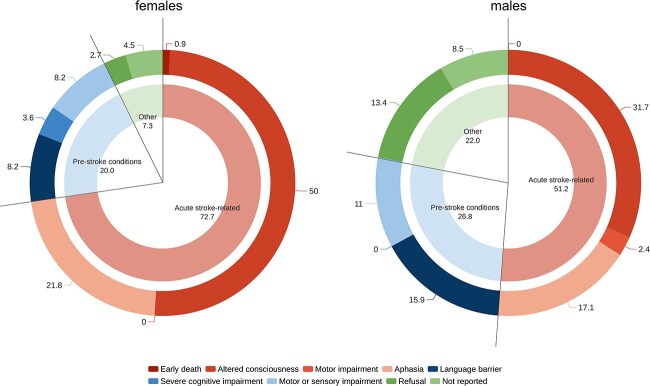
Sex-related differences in reasons for non-applicability of MoCA in the acute phase of stroke. The chart depicts the 110 female (A) and the 82 male (B) patients who were deemed not testable. Numbers in the chart represent percentages. Abbreviation: MoCA = Montreal Cognitive Assessment.

The MoCA was not applicable in 110 out of 291 (37.8%) females and in 82 out of 346 (23.7%) males (*P* <.001). Table 3 and Figure 2 show the sex-related differences for the non-applicability of the MoCA. Acute stroke-related reasons were more common among females (71.7% vs 51.2%, *P* =.002), as was altered consciousness (50.0% vs 31.7%, *P* =.011). Refusal of testing was more frequent among males (13.4% vs 2.7%, *P* =.005).

### 3.3. Baseline characteristics of patients tested and non-tested with MoCA

Table 1 shows the results of univariate analyses comparing baseline clinical variables in patients non-tested vs tested with MoCA. Compared to the tested group, those who did not undergo the MoCA were older (median age 82.6 vs 77.1 years, *P* <.001), less frequently male (42.7% vs 59.3%, *P* <.001), had lower education (mean education level 8.8 vs 9.9 years, *P* =.004), higher rates of pre-stroke cognitive impairment (45.5% vs 28.7%) and higher median NIHSS scores on admission (10.0 vs 2.0, *P* <.001).

Multivariable logistic regression analyses identified significant predictors for MoCA non-applicability (Table 4). In the first model on the whole sample, pre-stroke mRS score (adjusted OR [adj.OR] 1.26, 95% CI, 1.01–1.57), NIHSS score on admission (OR 1.23, 95% CI, 1.17–1.30) and left hemisphere lesion (OR 2.16, 95% CI, 1.29–3.62) were significantly associated with the non-applicability of MoCA, while sex was not. We also performed a sequential logistic regression analysis using MoCA non-applicability as the outcome variable, including sex as a fixed covariate and progressively adding other covariates. The association between sex and MoCA applicability lost statistical significance after the inclusion of NIHSS score in the model (adj.OR for sex 1.06, 95% CI, 0.63–1.78; adj.OR for NIHSS 1.23, 95% CI, 1.17–1.29).

Concerning sex-related differences, NIHSS score at onset was associated with MoCA non-applicability in both women (adj.OR 1.25, 95% CI, 1.16–1.34) and men (adj.OR 1.24, 95% CI, 1.14–1.34). Pre-stroke mRS was associated with non-applicability of MoCA in women (adj.OR 1.58, 95% CI, 1.15–2.17). Years of education and left hemisphere lesion were associated with non-applicability of MoCA in men (adj.OR 0.91, 95% CI, 0.84–1.00 and adj.OR 2.38, 95% CI, 1.16–4.86, respectively).

We performed sensitivity analyses excluding the 12 patients in whom the reason for MoCA non-applicability was not recorded. In *Model 1*, years of education reached statistical significance (adj.OR 1.06, 95% CI, 1.00–1.13), while the other variables remained unchanged. In *Model 2*, left hemisphere lesion reached statistical significance (adj.OR 0.43, 95% CI, 0.19–0.97), with no other changes observed. *Model 3* remained unchanged.

### 3.4. Sex-related MoCA performance in the acute stroke phase

**Figure 3:**
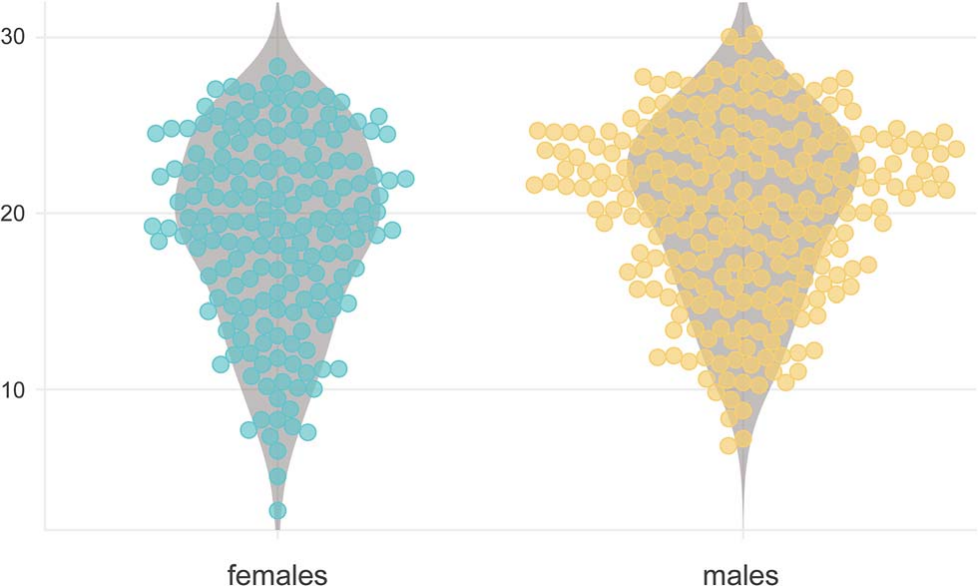
Sex-related differences in adjusted MoCA performances in the acute phase of stroke. The chart presents age and education-adjusted MoCA scores (*y* axis) for 175 female patients (A) and 255 male patients (B). Abbreviation: MoCA = Montreal Cognitive Assessment.

Among patients tested, the median raw MoCA score was 18 (IQR 13–23) in the total sample (*n* = 445) and the median adjusted MoCA score (*n* = 430) was 20.5 (IQR 16.0–23.9). Females had significantly lower raw MoCA scores than males (16 [IQR 10.5–23] vs 20 [IQR 15–23], *P* <.001). Median adjusted MoCA score was also lower in females than in males (19.5 [IQR 15.0–23.1] vs 21.1 [IQR 16.5–24.1], *P* =.022) (Figure 3).

We performed sensitivity analyses excluding patients with partially completed MoCA assessments (*n* = 69). In this subgroup, the raw MoCA score was different between females and males (17.0 [IQR 13.0–24.0] vs 21.0 [IQR 17.0–24.0], *P* =.001), while the adjusted MoCA score was similar between the two sexes (20.5 [IQR 16.9–24.3] in females vs 21.6 [IQR 17.8–24.4] in males, *P* =.081).

**Table 3:** Primary reasons for the non-applicability of the MoCA in female and male patients

**Reason for non-applicability of MoCA**	**All** ***N* = 192**	**Female** ***N* = 110**	**Male** ***N* = 82**	***P***-value
Acute stroke-related	122 (63.5)	80 (72.7)	42 (51.2)	**.002**
*Early death*	1 (0.5)	1 (0.9)	0 (0.0)	-
*Altered consciousness*	81 (42.2)	55 (50.0)	26 (31.7)	**.011**
*Motor deficit*	2 (1.0)	0 (0.0)	2 (2.4)	-
*Aphasia*	38 (19.8)	24 (21.8)	14 (17.1)	.414
Pre-stroke conditions	44 (22.9)	22 (20.0)	22 (26.8)	.265
*Language barrier*	22 (11.5)	9 (8.2)	13 (15.9)	.099
*Severe cognitive impairment*	4 (2.1)	4 (3.6)	0 (0.0)	-
*Motor or sensory impairment*	18 (9.4)	9 (8.2)	9 (11.0)	.511
Other	26 (13.5)	8 (7.3)	18 (22.0)	**.003**
*Refusal*	14 (7.3)	3 (2.7)	11 (13.4)	**.005**
*Reason not reported*	12 (6.3)	5 (4.5)	7 (8.5)	.258

Bold values represent statistically significant differences. Abbreviation: MoCA = Montreal Cognitive Assessment.

## 4. Discussion

In a cohort of patients hospitalised for an acute cerebrovascular event, we assessed the reasons and predictors of non-applicability of early cognitive screening with MoCA, focusing on sex-related differences. Females were less likely to receive cognitive screening in the acute phase, but they were older and had more severe strokes and higher levels of pre-stroke disability and cognitive impairment compared to males. Acute stroke-related reasons for the non-applicability of MoCA were more common among females, while refusal was more common in males. We identified sex-specific predictors of MoCA non-applicability: pre-stroke functional status in women, education level and left hemisphere lesions in men. Finally, regarding cognitive performance during the acute phase of stroke, we observed significantly lower raw and adjusted MoCA scores in females compared to males.

**Table 4:** Multivariable prediction models using baseline clinical variables as covariates and MoCA non-applicability as the outcome variable

**Covariate**	**Odds ratio (95% CI)**	***P*-value**
**(A) Model 1: all patients**		
Male sex	1.05 (0.61–1.80)	.852
Age	1.00 (0.97–1.02)	.870
Years of education	0.95 (0.89–1.01)	.078
Pre-stroke mRS score	1.26 (1.01–1.57)	**.039**
Pre-stroke cognitive impairment	1.19 (0.63–2.23)	.591
NIHSS score on admission	1.23 (1.17–1.30)	**<.001**
Left hemisphere lesion	2.16 (1.29–3.62)	**.002**
**(B) Model 2: females**		
Age	1.01 (0.97–1.06)	.542
Years of education	0.99 (0.90–1.08)	.757
Pre-stroke mRS score	1.58 (1.15–2.17)	**.005**
Pre-stroke cognitive impairment	1.18 (0.46–2.99)	.729
NIHSS score on admission	1.25 (1.16–1.34)	**<.001**
Left hemisphere lesion	2.10 (0.96–4.61)	.063
**(C) Model 3: males**		
Age	0.99 (0.96–1.02)	.540
Years of education	0.91 (0.84–1.00)	**.042**
Pre-stroke mRS score	0.94 (0.66–1.33)	.715
Pre-stroke cognitive impairment	1.09 (0.44–2.68)	.847
NIHSS score on admission	1.24 (1.14–1.34)	**<.001**
Left hemisphere lesion	2.38 (1.16–4.86)	**.018**

Bold values represent statistically significant differences. Abbreviations: MoCA = Montreal Cognitive Assessment; NIHSS = National Institutes of Health Stroke Scale; mRS = modified Rankin Scale; 95% CI = 95% confidence interval.

This study represents an effort to explore sex-related differences in the applicability and interpretation of early cognitive screening in an acute stroke setting. Nearly one in three patients was deemed ineligible for screening with MoCA, with females being less likely to undergo testing than males. Females also had lower rates of full MoCA completion compared to males. Nevertheless, sex was not associated with MoCA applicability after adjusting for confounding factors like stroke severity and pre-stroke disability, and this supports the robustness of this cognitive screening tool. Moreover, a sequential logistic regression analysis approach revealed that the association between sex and cognitive screening applicability was no longer significant after adjusting for stroke severity at onset, as measured by NIHSS. This finding reinforces the notion that stroke severity constitutes the main obstacle to conducting early cognitive screening in patients with acute cerebrovascular events. MoCA applicability appeared to differ across ischemic stroke subtypes, being lower in cardioembolic strokes and higher in small vessel occlusions, likely reflecting differences in stroke severity and language involvement.^23^ Given that cardioembolic strokes were more common among female patients in our sample, this factor may contribute—at least in part—to the observed sex-related differences in cognitive screening applicability. It is well-documented that females experience higher mortality and worse outcomes after stroke, a disparity partially attributed to more frequent misdiagnoses or delays in treatment.^24–26^ Additionally, females often present with stroke at an older age and with more comorbidities, complicating both acute management and long-term care.^12^ Similar to other sex-related differences observed in stroke outcomes, the lower applicability of MoCA in females seen in our study may be largely explained by differences in baseline functional and risk profiles between the sexes.^27^ Hence, further research is needed to explore the complexity of sex-related differences in the performance and utility of early cognitive screening in the acute stroke setting.

The reasons for the non-applicability of the MoCA significantly differed between females and males, with females more likely to have an acute stroke-related cause, primarily altered consciousness. Impaired consciousness has been frequently reported as one of the main reasons for the non-applicability of acute cognitive screening in previous studies.^11^^,^^28^ Females tend to present more often with disorders of consciousness at stroke onset, a condition associated with more severe symptoms and higher mortality.^25^^,^^29^ However, the expected utility of cognitive screening in patients with impaired consciousness appears negligible. Conversely, aphasia was the reason for MoCA non-applicability in about one in five patients, with no significant differences between sexes. Since cognitive screening tests—MoCA included—typically rely on verbal abilities, it is not surprising that aphasia was the primary reason for the non-applicability of acute screening in up to 60% of patients in previous studies.^11^^,^^28^ The use of cognitive screening tools that are less reliant on verbal abilities might help expand the applicability of such tests in acute stroke settings.^30^ Regarding non-acute stroke-related reasons for MoCA non-applicability, we observed that male patients were more likely to refuse cognitive screening. Previous evidence suggested the existence of sex-related differences in willingness to undergo cognitive testing, and further research is needed to identify strategies to improve patient engagement and commitment.^31^

We observed that baseline variables predicting MoCA applicability in the acute stroke phase differed between males and females. However, stroke severity at admission was consistently associated with the non-applicability of cognitive screening in both sexes. A left hemisphere lesion was associated with MoCA non-applicability across the entire sample and specifically in males, while only a trend was observed in females; this may probably be explained by a greater impact of impaired consciousness in females. Lower education levels predicted MoCA non-applicability in males but not in females. Less educated patients have been shown to be less willing to undergo cognitive testing, a challenge that could potentially be addressed by improving clinician-patient communication.^32^ Conversely, worse pre-stroke functional status (assessed through the mRS scale) was associated with non-applicability of cognitive screening in females but not in males. This could be linked to the poorer overall pre-morbid functional and cognitive status of female patients admitted to the stroke unit, which may prevent cognitive screening even in less severe strokes. As the population ages, many stroke patients are already living with dementia or disability at the time of their stroke, highlighting the need to determine the best diagnostic and management strategies for these frail patients.^15^

Regarding cognitive performance during the acute phase of stroke, we observed significantly lower raw and adjusted MoCA scores in females compared to males. However, this difference in adjusted scores appeared less pronounced in the sensitivity analysis excluding patients who did not fully complete the assessment, suggesting that completion rate may account for a substantial portion of the observed variability in MoCA performance. These findings further underscore the baseline disparities between female and male patients hospitalised for acute stroke, with women presenting more often with severe strokes, being generally older, and having lower levels of education.^25^^,^^33^ Given that MoCA performance in the acute phase has been shown to strongly predict long-term cognitive outcomes, our results highlight the need for personalised, longitudinal cognitive follow-up strategies.^6^^,^^34^^,^^35^ Such an approach should take into account potential sex-related differences that may already be evident in the acute phase.

The main strengths of our study are the large number of consecutive patients, the absence of exclusion criteria reflecting a real-world stroke population and the thorough evaluation of pre-stroke cognitive and functional status. However, we acknowledge that the single-center design limits the generalisability of our findings. Additionally, at present we did not investigate the long-term cognitive and functional trajectories of patients who were tested or not tested during the acute stroke phase. We also recognise that the absence of data regarding the reasons for MoCA non-applicability in a small subset of patients (6%) may have introduced a bias in the interpretation of our results. A further limitation is that, in patients for whom the MoCA was not applicable, we did not record the specific timing of the attempted assessment; however, in clinical practice, MoCA administration was generally attempted within the first 2-5 days of hospitalisation. Future studies should incorporate longitudinal, multicenter data and explore alternative cognitive assessment tools that are suitable for patients with severe impairments.

In conclusion, we observed sex-specific factors that might influence the feasibility and the interpretation of early cognitive screening using the MoCA in acute stroke patients. Further research is needed to determine the best strategies for conducting cognitive testing in the acute stroke setting.

## Supplementary Material

aakaf019_Sex-related_differences_in_MoCA_applicability_and_performance_checklist

## Data accessibility

Data that support the findings of this study are available from the corresponding author on reasonable request.

## References

[1] Pendlebury ST, Rothwell PM, Oxford Vascular Study. Incidence and prevalence of dementia associated with transient ischaemic attack and stroke: analysis of the population-based Oxford vascular study. Lancet Neurol. 2019;18:248-258. 10.1016/S1474-4422(18)30442-3 30784556 PMC6390174

[2] Mijajlović MD, Pavlović A, Brainin M, et al. Post-stroke dementia - a comprehensive review. BMC Med. 2017;15:11. 10.1186/s12916-017-0779-7 28095900 PMC5241961

[3] Quinn TJ, Elliott E, Langhorne P. Cognitive and mood assessment tools for use in stroke. Stroke. 2018;49:483-490. 10.1161/STROKEAHA.117.016994 29284733

[4] El Husseini N, Katzan IL, Rost NS, et al. Cognitive impairment after ischemic and Hemorrhagic stroke: a scientific statement from the American Heart Association/American Stroke Association. Stroke. 2023;54:e272-e291. 10.1161/STR.0000000000000430 37125534 PMC12723706

[5] Quinn TJ, Richard E, Teuschl Y, et al. European stroke organisation and European academy of neurology joint guidelines on post-stroke cognitive impairment. Eur Stroke J. 2021;6:I-XXXVIII. 10.1177/23969873211042192 PMC8564156 34746430

[6] Salvadori E, Cova I, Mele F, Pomati S, Pantoni L. Prediction of post-stroke cognitive impairment by Montreal cognitive assessment (MoCA) performances in acute stroke: comparison of three normative datasets. Aging Clin Exp Res. 2022;34:1855-1863. 10.1007/s40520-022-02133-9 35441928 PMC9283135

[7] Chiti G, Pantoni L. Use of Montreal cognitive assessment in patients with stroke. Stroke. 2014;45:3135-3140. 10.1161/STROKEAHA.114.004590 25116881

[8] Nasreddine ZS, Phillips NA, Bédirian V, et al. The Montreal cognitive assessment, MoCA: a brief screening tool for mild cognitive impairment. J Am Geriatr Soc. 2005;53:695-699. 10.1111/j.1532-5415.2005.53221.x 15817019

[9] Demeyere N . Acute post-stroke screening for a cognitive care pathway. Lancet Healthy Longev. 2024;5:e4-e5. 10.1016/S2666-7568(23)00257-X 38101425

[10] Lees RA, Hendry BAK, Broomfield N, et al. Cognitive assessment in stroke: feasibility and test properties using differing approaches to scoring of incomplete items. Int J Geriatr Psychiatry. 2017;32:1072-1078. 10.1002/gps.4568 27526678

[11] Pasi M, Salvadori E, Poggesi A, Inzitari D, Pantoni L. Factors predicting the Montreal cognitive assessment (MoCA) applicability and performances in a stroke unit. J Neurol. 2013;260:1518-1526. 10.1007/s00415-012-6819-5 23292208

[12] Phan HT, Reeves MJ, Blizzard CL, et al. Sex differences in severity of stroke in the INSTRUCT study: a meta-analysis of individual participant data. Journal of the American Heart Association: Cardiovascular and Cerebrovascular Disease. 2019;8:e010235. 10.1161/JAHA.118.010235 PMC6405721 30590965

[13] Appelros P, Åsberg S. Sex differences in stroke. Handb Clin Neurol. 2020;175:299-312. 10.1016/B978-0-444-64123-6.00021-7 33008533

[14] Strong B, Pudar J, Thrift AG, et al. Sex disparities in Enrollment in recent randomized clinical trials of acute stroke: a meta-analysis. JAMA Neurol. 2021;78:666-677. 10.1001/jamaneurol.2021.0873 33900363 PMC8077045

[15] Mele F, Cova I, Nicotra A, et al. Prestroke cognitive impairment: frequency and association with premorbid neuropsychiatric, functional, and neuroimaging features. Stroke. 2024;55:1869-1876. 10.1161/STROKEAHA.123.045344 38818731 PMC11198949

[16] Cova I, Mele F, Nicotra A, et al. The Luigi Sacco Hospital VAS-COG stroke care pathway: a five-year experience. Cereb Circ Cogn Behav. 2024;6:100210. 10.1016/j.cccb.2024.100210 38357360 PMC10865214

[17] Amarenco P, Bogousslavsky J, Caplan LR, Donnan GA, Hennerici MG. New approach to stroke subtyping: the A-S-C-O (phenotypic) classification of stroke. Cerebrovasc Dis. 2009;27:502-508. 10.1159/000210433 19342826

[18] Van Swieten JC, Koudstaal PJ, Visser MC, et al. Interobserver agreement for the assessment of handicap in stroke patients. Stroke. 1988;19:604-607. 10.1161/01.STR.19.5.604 3363593

[19] Jorm AF . A short form of the informant questionnaire on cognitive decline in the elderly (IQCODE): development and cross-validation. Psychol Med. 1994;24:145-153. 10.1017/S003329170002691X 8208879

[20] Aiello EN, Gramegna C, Esposito A, et al. The Montreal cognitive assessment (MoCA): updated norms and psychometric insights into adaptive testing from healthy individuals in northern Italy. Aging Clin Exp Res. 2022;34:375-382. 10.1007/s40520-021-01943-7 34313961 PMC8847194

[21] Suda S, Muraga K, Ishiwata A, et al. Early cognitive assessment following acute stroke: feasibility and comparison between mini-mental state examination and Montreal cognitive assessment. J Stroke Cerebrovasc Dis. 2020;29:104688. 10.1016/j.jstrokecerebrovasdis.2020.104688 32063455

[22] von Elm E, Altman DG, Egger M, et al. The strengthening the reporting of observational studies in epidemiology (STROBE) statement: guidelines for reporting observational studies. Lancet. 2007;370:1453-1457 18064739 10.1016/S0140-6736(07)61602-X

[23] Kamel H, Healey JS. Cardioembolic stroke. Circ Res. 2017;120:514-526. 10.1161/CIRCRESAHA.116.308407 28154101 PMC5312810

[24] Yu AYX, Penn AM, Lesperance ML, et al. Sex differences in presentation and outcome after an acute transient or minor neurologic event. JAMA Neurol. 2019;76:962-968. 10.1001/jamaneurol.2019.1305 31114842 PMC6537759

[25] Ali M, van Os HJA, van der Weerd N, et al. Sex differences in presentation of stroke: a systematic review and meta-analysis. Stroke. 2022;53:345-354. 10.1161/STROKEAHA.120.034040 34903037 PMC8785516

[26] Ali M, Dekker L, Daems JD, et al. Sex differences in prehospital identification of large vessel occlusion in patients with suspected stroke. Stroke. 2024;55:548-554. 10.1161/STROKEAHA.123.044898 38299328 PMC10896195

[27] Gall SL, Donnan G, Dewey HM, et al. Sex differences in presentation, severity, and management of stroke in a population-based study. Neurology. 2010;74:975-981. 10.1212/WNL.0b013e3181d5a48f 20181922

[28] Horstmann S, Rizos T, Rauch G, Arden C, Veltkamp R. Feasibility of the Montreal cognitive assessment in acute stroke patients. Eur J Neurol. 2014;21:1387-1393. 10.1111/ene.12505 25040216

[29] Weir CJ, Bradford APJ, Lees KR. The prognostic value of the components of the Glasgow coma scale following acute stroke. QJM. 2003;96:67-74. 10.1093/qjmed/hcg008. 12509651

[30] Yan Z, Xu S, Wei D, et al. Comparison of three cognitive assessment methods in post-stroke aphasia patients. Front Psychol. 2022;13:896095. 10.3389/fpsyg.2022.896095 36337480 PMC9631299

[31] Wong S, Jacova C. Older adults’ attitudes towards cognitive testing: moving towards person-centeredness. Dement Geriatr Cogn Dis Extra. 2018;8:348-359. 10.1159/000493464 30483302 PMC6243915

[32] Fowler NR, Perkins AJ, Turchan HA, et al. Older primary care patients’ attitudes and willingness to screen for dementia. J Aging Res. 2015;2015:1-7. 10.1155/2015/423265 PMC4417947 25973274

[33] Ospel J, Singh N, Ganesh A, Goyal M. Sex and gender differences in stroke and their practical implications in acute care. J Stroke. 2023;25:16-25. 10.5853/jos.2022.04077 36746379 PMC9911850

[34] Salvadori E, Pasi M, Poggesi A, Chiti G, Inzitari D, Pantoni L. Predictive value of MoCA in the acute phase of stroke on the diagnosis of mid-term cognitive impairment. J Neurol. 2013;260:2220-2227. 10.1007/s00415-013-6962-7 23716072

[35] Cova I, Mele F, Zerini F, et al. Neuropsychological screening in the acute phase of cerebrovascular diseases. Acta Neurol Scand. 2020;142:377-384. 10.1111/ane.13319 32687600

